# Supplementary material for: Virtual photons in the ground state of a dissipative system
Source: Nat Commun. 2017 Nov 13;8:1465. doi: 10.1038/s41467-017-01504-5 (PMC5684391; doi:10.1038/s41467-017-01504-5)
Supplement: Supplementary file 1 — Supplementary Information [file 41467_2017_1504_MOESM1_ESM.pdf]

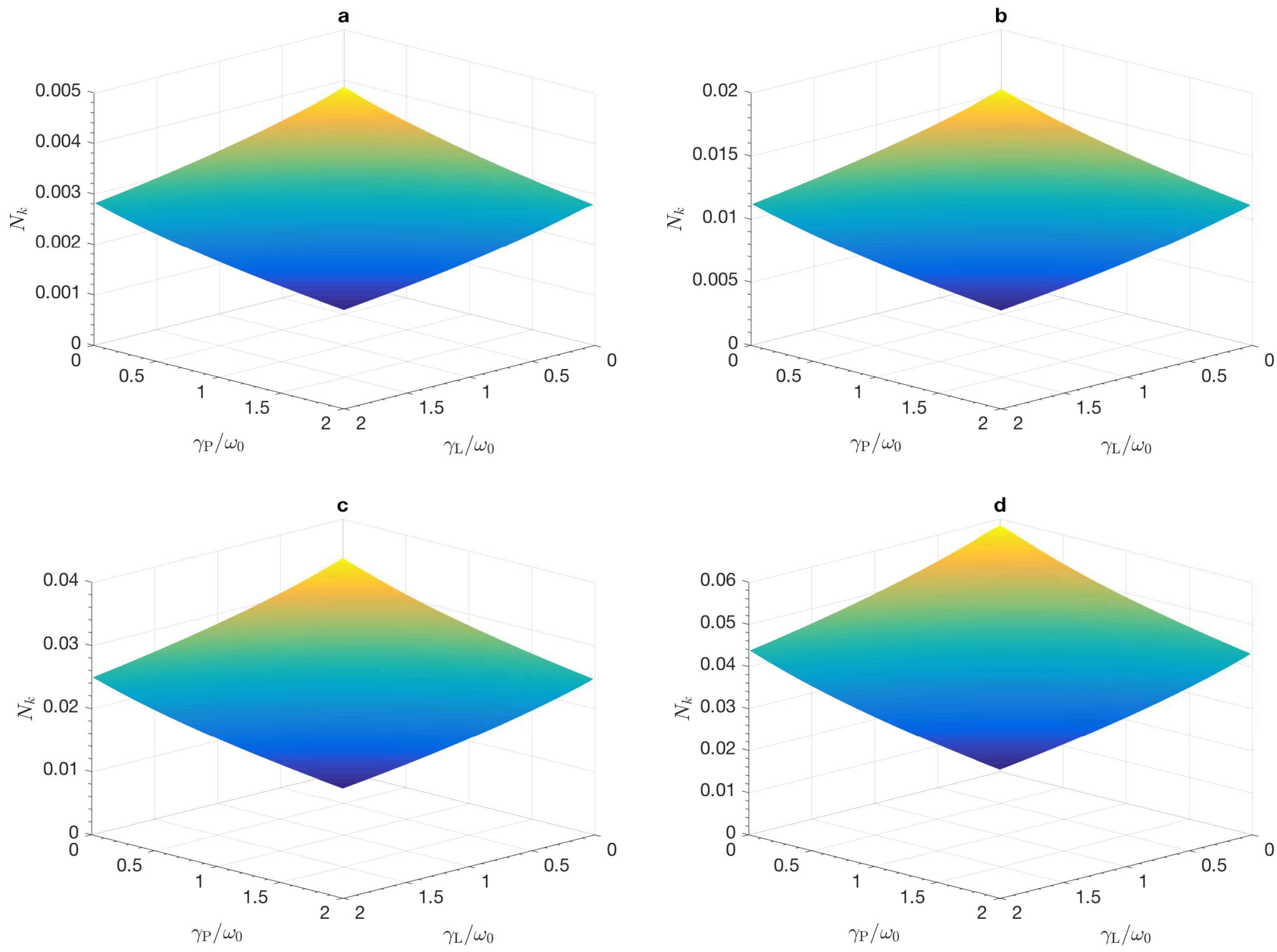

Supplementary Fig. 1: Virtual photon population for arbitrary losses. Number of virtual photons in the resonant mode $ck = \omega_0$ as a function of the matter (γ_{L}) and photonic (γ_{P}) losses for different values of the light-matter coupling ω_{c}. **a** $\omega_{\mathrm{c}} = 0.25\omega_0$. **b** $\omega_{\mathrm{c}} = 0.5\omega_0$. **c** $\omega_{\mathrm{c}} = 0.75\omega_0$. **d** $\omega_{\mathrm{c}} = \omega_0$.

Supplementary Note 1: Diagonalization of a dispersive-dissipative dielectric

In their seminal work [1] Huttner and Barnett extended the microscopic quantum theory of light-matter interaction developed by Hopfield [2] to the case of homogeneous but lossy dielectrics. They accomplished this by quantising the electromagnetic field coupled to a dispersionless matter excitation, itself coupled to a continuum reservoir of harmonic oscillators acting as a bath in which energy could be dissipated. The full Hamiltonian of such a system can be written as

$$\hat{H} \;=\; \hat{H}_{\mathrm{em}} + \hat{H}_{\mathrm{mat}} + \hat{H}_{\mathrm{int}} + \hat{H}_{\mathbf{A}^2}, \tag{1}$$

where

$$\hat{H}_{\mathrm{em}} = \sum_{\mathbf{k}} \hbar ck\, \hat{a}^{\dagger}(\mathbf{k})\hat{a}(\mathbf{k}), \tag{2}$$

describes the free electromagnetic field,

$$\hat{H}_{\mathrm{mat}} = \sum_{\mathbf{k}} \left\{ \hbar\tilde{\omega}_0\, \hat{b}^{\dagger}(\mathbf{k})\hat{b}(\mathbf{k}) + \int_0^{\infty} d\omega\; \hbar\omega\, \hat{b}^{\dagger}_{\omega}(\mathbf{k})\hat{b}_{\omega}(\mathbf{k}) + \int_0^{\infty} d\omega\; \frac{\hbar V(\omega)}{2} \left[\hat{b}^{\dagger}(-\mathbf{k}) + \hat{b}(\mathbf{k})\right] \left[\hat{b}^{\dagger}_{\omega}(\mathbf{k}) + \hat{b}_{\omega}(-\mathbf{k})\right] \right\}, \tag{3}$$

models the matter excitation and the bath,

$$\hat{H}_{\text{int}} = i\sum_{\mathbf{k}} \frac{\hbar\omega_{\text{c}}}{2}\sqrt{\frac{\tilde{\omega}_0}{ck}} \left[\hat{a}^\dagger(-\mathbf{k}) + \hat{a}(\mathbf{k})\right] \left[\hat{b}^\dagger(\mathbf{k}) - \hat{b}(-\mathbf{k})\right], \tag{4}$$

is the dipolar interaction between light and matter, and

$$\hat{H}_{\mathbf{A}^2} = \sum_{\mathbf{k}} \frac{\hbar\omega_{\text{c}}^2}{4ck} \left[\hat{a}^\dagger(-\mathbf{k}) + \hat{a}(\mathbf{k})\right] \left[\hat{a}^\dagger(\mathbf{k}) + \hat{a}(-\mathbf{k})\right], \tag{5}$$

comes from the diamagnetic $\mathbf{A}^2$ part of the the minimal-coupling Hamiltonian. In Supplementary Eqs. (2)-(5) $\hat{a}(\mathbf{k})$, $\hat{b}(\mathbf{k})$, and $\hat{b}_\omega(\mathbf{k})$, are bosonic annihilation operators respectively for a photon, a matter excitation, and an excitation of the bath with frequency ω, all indexed by the wavevector $\mathbf{k}$, ω_0 is the frequency of the optically active transition, $V(\omega)$ models its coupling to the bath, ω_{c} quantifies the intensity of the light-matter coupling, and the renormalised frequency $\tilde{\omega}_0$ is linked to the bare one by the formula

$$\tilde{\omega}_0^2 = \omega_0^2 + \int_0^\infty \frac{|V(\omega)|^2\tilde{\omega}_0}{\omega} d\omega. \tag{6}$$

Note that we can chose arbitrary large values for the light-matter coupling ω_{c} because, thanks to the presence of the term $\hat{H}_{\mathbf{A}^2}$, the ground state is stable and no superradiant phase transition can take place, as demonstrated by various no-go theorems [4, 5].

In Ref. [1] the authors make the choice to remove $\hat{H}_{\mathbf{A}^2}$ by effectively performing a Bogoliubov rotation in the space of the $\hat{a}(\mathbf{k})$, thus putting $\hat{H}_{\text{em}} + \hat{H}_{\mathbf{A}^2}$ in diagonal form in terms of the Bogoliubov-rotated operators $\hat{\bar{a}}(\mathbf{k})$

$$\hat{\bar{H}}_{\text{em}} = \sum_{\mathbf{k}} \hbar c\bar{k}\, \hat{\bar{a}}^\dagger(\mathbf{k})\hat{\bar{a}}(\mathbf{k}), \tag{7}$$

with $c\bar{k} = \sqrt{c^2k^2 + \omega_{\text{c}}^2}$. Unluckily this is not acceptable for us, because it implies that the very definition of the the bare photonic operators depends upon the strength of the coupling, and as such it becomes problematic to define the number of virtual photons in the ground state. Ignoring this problem leads to a number of inconsistencies, most notably the total energy per mode diverges for $k \to 0$. In the following we will thus sketch a derivation that keeps $\hat{H}_{\mathbf{A}^2}$ as part of the interaction. The Hamiltonian in Supplementary Eq. (1) can be diagonalised *a la* Fano [3] in two steps. First $\hat{H}_{\text{mat}}$ is put into diagonal form

$$\hat{H}_{\text{mat}} = \sum_{\mathbf{k}} \int_0^\infty d\omega\, \hbar\omega\, \hat{B}^\dagger(\mathbf{k},\omega)\hat{B}(\mathbf{k},\omega), \tag{8}$$

where the $\hat{B}(\mathbf{k},\omega)$ operators, describing the continuously broadened optically active matter resonance, obey bosonic commutation relations

$$\left[\hat{B}(\mathbf{k},\omega), \hat{B}^\dagger(\mathbf{k}',\omega')\right] = \delta(\mathbf{k}-\mathbf{k}')\delta(\omega-\omega'). \tag{9}$$

They can be expressed as linear combinations of the different uncoupled matter operators as

$$\hat{B}(\mathbf{k},\omega) = \alpha_0(\omega)\hat{b}(\mathbf{k}) + \beta_0(\omega)\hat{b}^\dagger(-\mathbf{k}) + \int_0^\infty d\omega' \left[\alpha_1(\omega,\omega')\hat{b}_{\omega'}(\mathbf{k}) + \beta_1(\omega,\omega')\hat{b}^\dagger_{\omega'}(-\mathbf{k})\right], \tag{10}$$

where the coefficients can be written as

$$\alpha_0(\omega) = \left(\frac{\omega+\tilde{\omega}_0}{2}\right)\frac{V(\omega)}{\omega^2 - \tilde{\omega}_0^2 z(\omega)}, \tag{11}$$

$$\beta_0(\omega) = \left(\frac{\omega-\tilde{\omega}_0}{2}\right)\frac{V(\omega)}{\omega^2 - \tilde{\omega}_0^2 z(\omega)}, \tag{12}$$

$$\alpha_1(\omega,\omega') = \delta(\omega-\omega') + \left(\frac{\tilde{\omega}_0}{2}\right)\left(\frac{V(\omega')}{\omega-\omega'-i0^+}\right)\frac{V(\omega)}{\omega^2 - \tilde{\omega}_0^2 z(\omega)}, \tag{13}$$

$$\beta_1(\omega,\omega') = \left(\frac{\tilde{\omega}_0}{2}\right)\left(\frac{V(\omega')}{\omega+\omega'}\right)\frac{V(\omega)}{\omega^2 - \tilde{\omega}_0^2 z(\omega)}, \tag{14}$$

and

$$z(\omega) = 1 - \frac{1}{2\tilde{\omega}_0}\left[\int_{-\infty}^{\infty} d\omega' \frac{|V(\omega')|^2}{\omega' - \omega + i0^+}\right]. \tag{15}$$

Introducing the quantity

$$\zeta(\omega) = i\sqrt{\tilde{\omega}_0}\left[\alpha_0(\omega) + \beta_0(\omega)\right] = i\frac{\sqrt{\tilde{\omega}_0}\omega V(\omega)}{\omega^2 - \tilde{\omega}_0^2 z(\omega)}, \tag{16}$$

which obeys the normalization

$$\int_0^{\infty} d\omega \frac{|\zeta(\omega)|^2}{\omega} \;=\; 1, \tag{17}$$

the interaction part of the Hamiltonian, now describing the interaction of the photonic modes with the broadened transition, takes the form

$$\hat{H}_{\text{int}} \;=\; \sum_{\mathbf{k}} \frac{\hbar\omega_{\text{c}}}{2\sqrt{ck}} \int_0^{\infty} d\omega \left\{\left[\zeta(\omega)\hat{B}^\dagger(\mathbf{k},\omega) + \zeta^*(\omega)\hat{B}(-\mathbf{k},\omega)\right]\left[\hat{a}^\dagger(-\mathbf{k}) + \hat{a}(\mathbf{k})\right]\right\}. \tag{18}$$

Analogously to the above, the novel Hamiltonian can now be put into diagonal form

$$\hat{H} = \sum_{\mathbf{k}} \int_0^{\infty} d\omega\; \hbar\omega\; \hat{C}^\dagger(\mathbf{k},\omega)\hat{C}(\mathbf{k},\omega), \tag{19}$$

through the operators

$$\hat{C}(\mathbf{k},\omega) = \tilde{\alpha}_{0,k}(\omega)\hat{a}(\mathbf{k}) + \tilde{\beta}_{0,k}(\omega)\hat{a}^\dagger(-\mathbf{k}) + \int_0^{\infty} d\omega' \;\left[\tilde{\alpha}_{1,k}(\omega,\omega')\hat{B}(\mathbf{k},\omega') + \tilde{\beta}_{1,k}(\omega,\omega')\hat{B}^\dagger(-\mathbf{k},\omega')\right], \tag{20}$$

with the coefficients

$$\tilde{\alpha}_{0,k}(\omega) \;=\; \sqrt{\frac{\omega_{\text{c}}^2}{ck}}\left(\frac{\omega + ck}{2}\right)\frac{\zeta(\omega)}{\epsilon^*(\omega)\omega^2 - c^2k^2}, \tag{21}$$

$$\tilde{\beta}_{0,k}(\omega) \;=\; \sqrt{\frac{\omega_{\text{c}}^2}{ck}}\left(\frac{\omega - ck}{2}\right)\frac{\zeta(\omega)}{\epsilon^*(\omega)\omega^2 - c^2k^2}, \tag{22}$$

$$\tilde{\alpha}_{1,k}(\omega,\omega') \;=\; \delta(\omega - \omega') + \frac{\omega_{\text{c}}^2}{2}\frac{\zeta^*(\omega')}{\omega - \omega' - i0^+}\frac{\zeta(\omega)}{\epsilon^*(\omega)\omega^2 - c^2k^2}, \tag{23}$$

$$\tilde{\beta}_{1,k}(\omega,\omega') \;=\; \frac{\omega_{\text{c}}^2}{2}\frac{\zeta(\omega')}{\omega + \omega'}\frac{\zeta(\omega)}{\epsilon^*(\omega)\omega^2 - c^2k^2}, \tag{24}$$

where the complex dielectric function is

$$\epsilon(\omega) = 1 + \frac{\omega_{\text{c}}^2}{2\omega}\int_{-\infty}^{\infty} d\omega' \frac{|\zeta(\omega')|^2}{\omega'\left(\omega' - \omega - i0^+\right)}. \tag{25}$$

In comparison the equivalent of the coefficient in Supplementary Eq. (21) obtained in Ref. [1] has instead the form

$$\tilde{\alpha}_{0,k}^{\text{HB}}(\omega) \;=\; \sqrt{\frac{\omega_{\text{c}}^2}{c\bar{k}}}\left(\frac{\omega + c\bar{k}}{2}\right)\frac{\zeta(\omega)}{\epsilon^*(\omega)\omega^2 - c^2k^2}, \tag{26}$$

with the others following a similar scheme.

Supplementary Note 2: Virtual photon population in the lossless case

In order to verify our calculations, it can be of use to compare the results obtained through the dissipative theory in the case of vanishing losses with those obtained using the standard nondissipative theory due to Hopfield [2]. In

order to do this we start from the equivalent of Supplementary Eq. (1) neglecting the bath

$$\hat{H}_{\rm Hop} = \sum_{\mathbf{k}} \Big\{ \hbar ck\, \hat{a}^\dagger(\mathbf{k})\hat{a}(\mathbf{k}) + \hbar\omega_0\, \hat{b}^\dagger(\mathbf{k})\hat{b}(\mathbf{k}) \tag{27}$$

$$+ i\frac{\hbar\omega_{\rm c}}{2}\sqrt{\frac{\omega_0}{ck}}\left[\hat{a}^\dagger(-\mathbf{k})+\hat{a}(\mathbf{k})\right]\left[\hat{b}^\dagger(\mathbf{k})-\hat{b}(-\mathbf{k})\right] + \frac{\hbar\omega_{\rm c}^2}{4ck}\left[\hat{a}^\dagger(-\mathbf{k})+\hat{a}(\mathbf{k})\right]\left[\hat{a}^\dagger(\mathbf{k})+\hat{a}(-\mathbf{k})\right]\Big\}.$$

The Hamiltonian in Supplementary Eq. (27) can be put in the diagonal form

$$\hat{H}_{\rm Hop} = \sum_{\mathbf{k}}\sum_{j=\pm} \hbar\omega_{j,k}\, \hat{p}_j^\dagger(\mathbf{k})\hat{p}_j(\mathbf{k}), \tag{28}$$

through the introduction of the polaritonic operators

$$\hat{p}_\pm(\mathbf{k}) = w_{\pm,k}\hat{a}(\mathbf{k}) + x_{\pm,k}\hat{b}(\mathbf{k}) + y_{\pm,k}\hat{a}^\dagger(-\mathbf{k}) + z_{\pm,k}\hat{b}^\dagger(-\mathbf{k}), \tag{29}$$

where

$$\omega_{\pm,k} = \sqrt{\frac{\omega_0^2+\omega_{\rm c}^2+c^2k^2 \pm \sqrt{(\omega_0^2+\omega_{\rm c}^2+c^2k^2)^2-4c^2k^2\omega_0^2}}{2}}, \tag{30}$$

and

$$\begin{pmatrix} w_{\pm,k} \\ x_{\pm,k} \\ y_{\pm,k} \\ z_{\pm,k} \end{pmatrix} = \pm\left\{\frac{\omega_{\pm,k}}{\omega_0}\left[\left(1-\frac{\omega_{\pm,k}^2}{\omega_0^2}\right)^2+\frac{\omega_{\rm c}^2}{\omega_0^2}\right]\right\}^{-\frac{1}{2}} \begin{pmatrix} \left[\frac{\omega_{\pm,k}^2}{\omega_0^2}-1\right]\frac{\omega_{\pm,k}+ck}{2\omega_0}\sqrt{\frac{\omega_0}{ck}} \\ i\frac{\omega_{\rm c}}{2\omega_0}(1+\frac{\omega_{\pm,k}}{\omega_0}) \\ \left[\frac{\omega_{\pm,k}^2}{\omega_0^2}-1\right]\frac{\omega_{\pm,k}-ck}{2\omega_0}\sqrt{\frac{\omega_0}{ck}} \\ i\frac{\omega_{\rm c}}{2\omega_0}(1-\frac{\omega_{\pm,k}}{\omega_0}) \end{pmatrix}. \tag{31}$$

The transformation from bare to polaritonic basis in Supplementary Eq. (29) can be inverted as

$$\hat{a}(\mathbf{k}) = w_{+,k}^*\hat{p}_+(\mathbf{k}) + w_{-,k}^*\hat{p}_-(\mathbf{k}) - y_{+,k}\hat{p}_+^\dagger(-\mathbf{k}) - y_{-,k}\hat{p}_-^\dagger(-\mathbf{k}), \tag{32}$$

$$\hat{b}(\mathbf{k}) = x_{+,k}^*\hat{p}_+(\mathbf{k}) + x_{-,k}^*\hat{p}_-(\mathbf{k}) - z_{+,k}\hat{p}_+^\dagger(-\mathbf{k}) - z_{-,k}\hat{p}_-^\dagger(-\mathbf{k}), \tag{33}$$

and the photonic population in the mode $\mathbf{k}$ calculated with the Hopfield theory is thus

$$N_k' = \sum_{j=\pm}|y_{j,k}|^2. \tag{34}$$

We can now write N_k in the limit of vanishing losses, and thus real eigenfrequencies, as

$$\lim_{V(\omega)\to 0} N_k = \sum_{j=\pm}\left[\frac{{\omega_{j,k}}^2-c^2k^2}{4c^3k^2}\frac{d\omega_{j,k}}{dk}\right]-\frac{1}{2}. \tag{35}$$

Plugging Supplementary Eqs. (30)-(31) into Supplementary Eqs. (34)-(35), and developing the heavy but straightforward algebra we can then prove that the two expressions coincide, verifying the correctness of our procedure.

Supplementary Note 3: Lorentz dielectric function

Here we will prove that the Lorentz dielectric function

$$\epsilon_{\rm L}(\omega) = 1 + \frac{\omega_{\rm c}^2}{\omega_0^2-\omega^2-i\gamma_{\rm L}\omega}, \tag{36}$$

used through the paper is consistent with the general form in Supplementary Eq. (25). We will do so by explicitly showing that the form in Supplementary Eq. (36) is recovered by evaluating Supplementary Eq. (25) with a coupling of the form

$$|V(\omega)|^2 = \frac{\omega\tilde{\omega}_0}{q+\omega_M}, \tag{37}$$

where ω_M is a cutoff frequency that we will eventually send to infinity and q a positive constant frequency. We will limit ourselves to consider the imaginary part of the dielectric function

$$\mathrm{Im}\left[\epsilon(\omega)\right] = \frac{\omega_{\mathrm{c}}^2\,\tilde{\omega}_0^2 \mathrm{Im}\left[z(\omega)\right]}{(\omega^2-\tilde{\omega}_0^2\mathrm{Re}\left[z(\omega)\right])^2+(\tilde{\omega}_0^2\mathrm{Im}\left[z(\omega)\right])^2}, \tag{38}$$

as the real part will be fixed by Kramers-Kronig relations. Plugging Supplementary Eq. (37) into Supplementary Eq. (6), and introducing the cutoff in the integration we obtain

$$\tilde{\omega}_0^2 = \omega_0^2 + \int_0^{\omega_M} \frac{|V(\omega)|^2\tilde{\omega}_0}{\omega} d\omega = \omega_0^2 \frac{q+\omega_M}{q}. \tag{39}$$

From Supplementary Eq. (15), using the Sokhotski-Plemelj theorem

$$\begin{aligned} \mathrm{Im}\left[z(\omega)\right] &= \frac{\pi}{2}\frac{|V(\omega)|^2}{\tilde{\omega}_0} = \frac{\pi}{2}\frac{\omega}{q+\omega_M}, &(40)\\ \mathrm{Re}\left[z(\omega)\right] &= 1-\frac{1}{2\tilde{\omega}_0}\mathcal{P}\int_{-\omega_M}^{\omega_M} d\omega' \frac{|V(\omega')|^2}{\omega'-\omega} = 1-\frac{\omega_M}{q+\omega_M}+\frac{\omega\log\left(1-\frac{2\omega}{\omega+\omega_M}\right)}{2(q+\omega_M)}, \end{aligned}$$

where the $\mathcal{P}$ indicates the principal part of the integral. Sending the cutoff to infinity we thus have

$$\lim_{\omega_M\to\infty}\tilde{\omega}_0^2\,\mathrm{Im}\left[z(\omega)\right] = \frac{\pi}{2}\frac{\omega_0^2}{q}\omega, \tag{41}$$

$$\lim_{\omega_M\to\infty}\tilde{\omega}_0^2\,\mathrm{Re}\left[z(\omega)\right] = \omega_0^2. \tag{42}$$

Using Supplementary Eqs. (41)-(42) into Supplementary Eq. (38) we finally get

$$\mathrm{Im}\left[\epsilon(\omega)\right] = \frac{\omega_{\mathrm{c}}^2\,\omega_0^2\frac{\omega}{q}\frac{\pi}{2}}{(\omega^2-\omega_0^2)^2+(\omega_0^2\frac{\omega}{q}\frac{\pi}{2})^2}, \tag{43}$$

which is the imaginary part of the Lorentz dielectric function

$$\mathrm{Im}\left[\epsilon_{\mathrm{L}}(\omega)\right] = \frac{\omega_{\mathrm{c}}^2\gamma_{\mathrm{L}}\omega}{(\omega_0^2-\omega^2)^2+\gamma_{\mathrm{L}}^2\omega^2}, \tag{44}$$

upon the identification

$$\gamma_{\mathrm{L}} = \frac{\pi\omega_0^2}{2q}. \tag{45}$$

Supplementary Note 4: Virtual photon population with arbitrary losses

In the general situation in which photons can be lost both through absorption and leakage, we need to consider a more general Hamiltonian

$$\hat{H}' = \hat{H}'_{\mathrm{em}} + \hat{H}_{\mathrm{mat}} + \hat{H}_{\mathrm{int}} + \hat{H}_{\mathbf{A}^2}, \tag{46}$$

with

$$\hat{H}'_{\mathrm{em}} = \sum_{\mathbf{k}}\left\{\hbar c\tilde{k}\,\hat{a}^\dagger(\mathbf{k})\hat{a}(\mathbf{k}) + \int_0^\infty d\omega\;\hbar\omega\,\hat{a}_\omega^\dagger(\mathbf{k})\hat{a}_\omega(\mathbf{k}) + \int_0^\infty d\omega\;\frac{\hbar Q_k(\omega)}{2}\left[\hat{a}^\dagger(\mathbf{k})+\hat{a}(-\mathbf{k})\right]\left[\hat{a}_\omega^\dagger(-\mathbf{k})+\hat{a}_\omega(\mathbf{k})\right]\right\}. \tag{47}$$

The Hamiltonian in Supplementary Eq. (47) is similar to the one in Supplementary Eq. (1) but the external bath, modelling in this case a continuum of photonic modes represented by bosonic operators $\hat{a}_\omega(\mathbf{k})$, is now coupled through the coupling $Q_k(\omega)$ to the photonic excitation whose renormalised frequency is given by

$$c^2\tilde{k}^2 = c^2k^2 + \int_0^\infty d\omega \frac{|Q_k(\omega)|^2 c\tilde{k}}{\omega}. \tag{48}$$

The Hamiltonian in Supplementary Eq. (47) can be put in the diagonal form

$$\hat{H}'_{\rm em} = \sum_{\mathbf{k}} \int_0^\infty d\omega \; \hbar\omega \, \hat{A}^\dagger(\mathbf{k},\omega)\hat{A}(\mathbf{k},\omega), \tag{49}$$

in terms of the operators describing the continuum spectrum of a leaky resonator

$$\hat{A}(\mathbf{k},\omega) = \breve{\alpha}_{0,k}(\omega)\hat{a}(\mathbf{k}) + \breve{\beta}_{0,k}(\omega)\hat{a}^\dagger(-\mathbf{k}) + \int_0^\infty d\omega' \; \left[\breve{\alpha}_{1,k}(\omega,\omega')\hat{a}_{\omega'}(\mathbf{k}) + \breve{\beta}_{1,k}(\omega,\omega')\hat{a}^\dagger_{\omega'}(-\mathbf{k})\right], \tag{50}$$

where the coefficients are

$$\breve{\alpha}_{0,k}(\omega) \;=\; \left(\frac{\omega + c\tilde{k}}{2}\right) \frac{Q_k(\omega)}{\omega^2 - c^2\tilde{k}^2 t(\omega)}, \tag{51}$$

$$\breve{\beta}_{0,k}(\omega) \;=\; \left(\frac{\omega - c\tilde{k}}{2}\right) \frac{Q_k(\omega)}{\omega^2 - c^2\tilde{k}^2 t(\omega)}, \tag{52}$$

$$\breve{\alpha}_{1,k}(\omega,\omega') \;=\; \delta(\omega-\omega') + \left(\frac{c\tilde{k}}{2}\right)\left(\frac{Q_k(\omega')}{\omega-\omega'-i0^+}\right)\frac{Q_k(\omega)}{\omega^2 - c^2\tilde{k}^2 t(\omega)}, \tag{53}$$

$$\breve{\beta}_{1,k}(\omega,\omega') \;=\; \left(\frac{c\tilde{k}}{2}\right)\left(\frac{Q_k(\omega')}{\omega+\omega'}\right)\frac{Q_k(\omega)}{\omega^2 - c^2\tilde{k}^2 t(\omega)}, \tag{54}$$

and

$$t(\omega) \;=\; 1 - \frac{1}{2c\tilde{k}}\left[\int_{-\infty}^{\infty} d\omega' \frac{|Q_k(\omega')|^2}{\omega'-\omega+i0^+}\right]. \tag{55}$$

Introducing the quantity

$$\chi_k(\omega) = \frac{1}{\sqrt{c\tilde{k}}}\left[\breve{\alpha}_{0,k}(\omega) - \breve{\beta}_{0,k}(\omega)\right] = \sqrt{c\tilde{k}}\frac{Q_k(\omega)}{\omega^2 - c^2\tilde{k}^2 t(\omega)}, \tag{56}$$

which obeys the normalization

$$\int_0^\infty d\omega \; \omega|\chi_k(\omega)|^2 \;=\; 1, \tag{57}$$

the interaction part of the Hamiltonian, describing two coupled continua, takes the form

$$\hat{H}_{\rm int} \;=\; \sum_{\mathbf{k}} \frac{\hbar\omega_{\rm c}}{2}\int_0^\infty d\omega \int_0^\infty d\omega' \left[\chi_k(\omega)\hat{A}^\dagger(-\mathbf{k},\omega) + \chi_k^*(\omega)\hat{A}(\mathbf{k},\omega)\right]\left[\zeta(\omega')\hat{B}^\dagger(\mathbf{k},\omega') + \zeta^*(\omega')\hat{B}(-\mathbf{k},\omega')\right], \tag{58}$$

$$\hat{H}_{\mathbf{A}^2} \;=\; \sum_{\mathbf{k}} \frac{\hbar\omega_{\rm c}^2}{4}\int_0^\infty d\omega \int_0^\infty d\omega' \left[\chi_k(\omega)\hat{A}^\dagger(\mathbf{k},\omega) + \chi_k^*(\omega)\hat{A}(-\mathbf{k},\omega)\right]\left[\chi_k(\omega')\hat{A}^\dagger(-\mathbf{k},\omega') + \chi_k^*(\omega')\hat{A}(\mathbf{k},\omega')\right]. \tag{59}$$

Contrary to the case treated previously, describing a single continuum coupled to a discrete resonance, the diagonalization of two coupled continua was not treated in the original paper by Fano [3] and it does not seem to be readily available in the literature. We will thus describe the procedure in more details. We aim to diagonalize the system in terms of two branches of continuum polaritonic modes

$$\hat{P}_\pm(\mathbf{k},\omega) = \int_0^\infty d\omega' \left[w_{\pm,k}(\omega,\omega')\hat{A}(\mathbf{k},\omega') + x_{\pm,k}(\omega,\omega')\hat{B}(\mathbf{k},\omega') + y_{\pm,k}(\omega,\omega')\hat{A}^\dagger(-\mathbf{k},\omega') + z_{\pm,k}(\omega,\omega')\hat{B}^\dagger(-\mathbf{k},\omega')\right], \tag{60}$$

obeying bosonic commutation relations

$$\left[\hat{P}_j(\mathbf{k},\omega), \hat{P}^\dagger_{j'}(\mathbf{k}',\omega')\right] = \delta(\mathbf{k}-\mathbf{k}')\delta(\omega-\omega')\delta_{jj'}. \tag{61}$$

The eigenequation

$$\omega\hat{P}_\pm(\mathbf{k},\omega) \;=\; [\hat{P}_\pm(\mathbf{k},\omega), \hat{H}'], \tag{62}$$

leads to the system

$$(\omega-\omega')w_{j,k}(\omega,\omega') = \omega_c^2\chi_k^*(\omega')\int d\omega''\frac{\omega''}{\omega+\omega''}w_{j,k}(\omega,\omega'')\chi_k(\omega'')+\omega_c\chi_k^*(\omega')\int d\omega''\frac{\omega''}{\omega+\omega''}x_{j,k}(\omega,\omega'')\zeta(\omega''), \quad (63)$$

$$(\omega-\omega')x_{j,k}(\omega,\omega') = \omega_c\zeta^*(\omega')\int d\omega''\frac{\omega''}{\omega+\omega''}w_{j,k}(\omega,\omega'')\chi_k(\omega''), \quad (64)$$

where the other two coefficients obey

$$(\omega-\omega')w_{j,k}(\omega,\omega')\chi_k(\omega') = (\omega+\omega')y_{j,k}(\omega,\omega')\chi_k^*(\omega'), \quad (65)$$

$$(\omega-\omega')x_{j,k}(\omega,\omega')\zeta(\omega') = (\omega+\omega')z_{j,k}(\omega,\omega')\zeta^*(\omega'). \quad (66)$$

Defining the functions

$$K_{j,k}(\omega) = \int d\omega'\frac{\omega'\chi_k(\omega')}{\omega+\omega'}w_{j,k}(\omega,\omega'), \quad (67)$$

$$W_k(\omega) = \mathcal{P}\int d\omega'\frac{\omega'|\chi_k(\omega')|^2}{\omega^2-\omega'^2}, \quad (68)$$

$$Z(\omega) = \mathcal{P}\int d\omega'\frac{\omega^2|\zeta(\omega')|^2}{\omega'(\omega^2-\omega'^2)}, \quad (69)$$

where $\mathcal{P}$ denotes the principal part, we can formally solve for the coefficients as

$$x_{j,k}(\omega,\omega')\zeta(\omega') = \left[\mathcal{P}\frac{1}{\omega-\omega'}+s_{x,j,k}(\omega)\delta(\omega-\omega')\right]\omega_c|\zeta(\omega')|^2K_{j,k}(\omega), \quad (70)$$

$$w_{j,k}(\omega,\omega')\chi_k(\omega') = \left[\mathcal{P}\frac{1}{\omega-\omega'}+s_{w,j,k}(\omega)\delta(\omega-\omega')\right]\left[\frac{s_{x,j,k}(\omega)|\zeta(\omega)|^2}{2}+Z(\omega)\right]\omega_c^2|\chi_k(\omega')|^2K_{j,k}(\omega), \quad (71)$$

with $s_{x,j,k}(\omega)$ and $s_{w,j,k}(\omega)$ functions to be determined. Multiplying Supplementary Eq. (71) by $\frac{\omega'}{\omega+\omega'}$, integrating, and exploiting Supplementary Eq. (67), we finally get to the equation

$$1 = \omega_c^2\left[\frac{s_{w,j,k}(\omega)|\chi_k(\omega)|^2}{2}+W_k(\omega)\right]\left[\frac{s_{x,j,k}(\omega)|\zeta(\omega)|^2}{2}+Z(\omega)\right]. \quad (72)$$

In order to fix the extra function introduced in Supplementary Eqs. (70)-(71) we can now exploit Supplementary Eq. (61), which after some algebra leads to

$$\Bigg[\omega_c^2|\chi_k(\omega)|^2\left[\pi^2+s^*_{w,j',k}(\omega)s_{w,j,k}(\omega)\right]\left[\frac{s_{x,j,k}(\omega)|\zeta(\omega)|^2}{2}+Z(\omega)\right]\left[\frac{s^*_{x,j',k}(\omega)|\zeta(\omega)|^2}{2}+Z(\omega)\right] \quad (73)$$
$$+|\zeta(\omega)|^2\left[\pi^2+s^*_{x,j',k}(\omega)s_{x,j,k}(\omega)\right]\Bigg]\omega_c^2K_{j,k}(\omega)K^*_{j',k}(\omega)=\delta_{jj'}.$$

We have at this point determined five equations, two from Supplementary Eq. (72) and three from Supplementary Eq. (73), in the six unknown functions $K_{j,k}(\omega), s_{w,j,k}(\omega), s_{x,j,k}(\omega)$, with $j=\pm$. The two eigenmodes $\hat{P}_\pm(\mathbf{k},\omega)$ are degenerate for each value of $\mathbf{k}$ and ω. This implies a basis in such a degenerate subspace needs to be chosen through gauge fixing, leading to a sixth equation which then allows to algebraically solve the system and complete the diagonalization. We will fix this gauge freedom by choosing the basis in which $w_{+,k}(\omega,\omega)=0$, which from Supplementary Eq. (71) implies $s_{w,+,k}=0$. The number of ground state photons can at this point be readily determined by the formula

$$N_k = \sum_{j=\pm}\int_0^\infty d\omega\int_0^\infty d\omega'|y_{j,k}(\omega,\omega')|^2=\sum_{j=\pm}\int_0^\infty d\omega'\frac{\omega_c^4|\chi_k(\omega')|^2}{(\omega+\omega')^2}\int_0^\infty d\omega\;|\frac{s_{x,j,k}(\omega)|\zeta(\omega)|^2}{2}+Z(\omega)|^2|K_{j,k}(\omega)|^2. \quad (74)$$

In order to get quantitative results from Supplementary Eq. (74) we now need to fix the coupling function $Q_k(\omega)$. The ultrastrong coupling regime has been achieved using many different kinds of photonic resonators, each one described by a different $Q_k(\omega)$. In order to achieve an acceptable level of generality, as done for the matter losses, we specialise the theory to the case of a Lorentzian resonance, which is usually an acceptable approximation for most real

resonators in a quite broad parameter range. We thus consider a coupling of the form of the one in Supplementary Eq. (37)

$$|Q_k(\omega)|^2 = \frac{\omega c\tilde{k}}{\tilde{q} + \omega_M}, \tag{75}$$

with

$$\tilde{q} = \frac{\pi c^2 k^2}{2\gamma_{\rm P}}. \tag{76}$$

Calculating all the relevant integrals, and letting $\omega_M \to \infty$ at the end, we recover the normalised density for the photonic mode with broadening $\gamma_{\rm P}$

$$\omega|\chi_k(\omega)|^2 = \frac{2\gamma_{\rm P}}{\pi} \frac{\omega^2}{(c^2k^2 - \omega^2)^2 + \gamma_{\rm P}^2\omega^2}. \tag{77}$$

In the Supplementary Fig. 1 we plot the virtual photonic population of the resonant mode as a function of $\gamma_{\rm L}$ and $\gamma_{\rm P}$ for different values of $\omega_{\rm c}$. We can clearly see that in the considered parameter range N_k essentially depends on the total broadening $\gamma_{\rm L} + \gamma_{\rm P}$, and that a sizeable virtual photon population, equal to roughly the 50% of the lossless one, remains even when $\gamma_{\rm L} = \gamma_{\rm P} = \gamma_{\rm max}$ and light and matter resonances are both overdamped.

Supplementary References

[1] B. Huttner and S. M. Barnett, *Quantization of the Electromagnetic Field in Dielectrics.* Phys. Rev. A **46**, 4306-4322 (1992).

[2] J. J. Hopfield, *Theory of the Contribution of Excitons to the Complex Dielectric Constant of Crystals.* Phys. Rev. **112**, 1555-1567 (1958).

[3] U. Fano, *Effects of configuration interaction on intensities and phase shifts.* Phys. Rev. **124**, 1866-1878 (1956).

[4] K. Rzaźewski, K Wodḱiewicz, and W. Źacowicz, *Phase transitions, two-level atoms, and the A^2 term.* Phys. Rev. Lett. **35**, 432-434 (1975).

[5] M. Bamba and T. Ogawa, *Stability of polarizable materials against superradiant phase transition.* Phys. Rev. A **90**, 063825 (2014).
